# Brain MR Contribution to the Differential Diagnosis of Parkinsonian Syndromes: An Update

**DOI:** 10.1155/2016/2983638

**Published:** 2016-09-28

**Authors:** Giovanni Rizzo, Stefano Zanigni, Roberto De Blasi, Daniela Grasso, Davide Martino, Rodolfo Savica, Giancarlo Logroscino

**Affiliations:** ^1^IRCCS Istituto delle Scienze Neurologiche, Bellaria Hospital, Bologna, Italy; ^2^Neurology Unit, Department of Biomedical and Neuromotor Sciences, University of Bologna, Bologna, Italy; ^3^Functional MR Unit, Policlinico S.Orsola-Malpighi, Bologna, Italy; ^4^Department of Biomedical and Neuromotor Sciences, University of Bologna, Bologna, Italy; ^5^Department of Diagnostic Imaging, Pia Fondazione di Culto e Religione “Card. G. Panico”, Tricase, Italy; ^6^Department of Neurology, King's College NHS Foundation Trust, London, UK; ^7^Department of Neurology, Queen Elizabeth Hospital, Lewisham and Greenwich NHS Trust, London, UK; ^8^Department of Neurology and Health Science Research, Mayo Clinic, Rochester, MN, USA; ^9^Department of Clinical Research in Neurology, University of Bari, Pia Fondazione di Culto e Religione “Card. G. Panico”, Tricase, Italy; ^10^Department of Basic Medical Science, Neuroscience and Sense Organs, University of Bari, Bari, Italy

## Abstract

Brain magnetic resonance (MR) represents a useful and feasible tool for the differential diagnosis of Parkinson's disease. Conventional MR may reveal secondary forms of parkinsonism and may show peculiar brain alterations of atypical parkinsonian syndromes. Furthermore, advanced MR techniques, such as morphometric-volumetric analyses, diffusion-weighted imaging, diffusion tensor imaging, tractography, proton MR spectroscopy, and iron-content sensitive imaging, have been used to obtain quantitative parameters useful to increase the diagnostic accuracy. Currently, many MR studies have provided both qualitative and quantitative findings, reflecting the underlying neuropathological pattern of the different degenerative parkinsonian syndromes. Although the variability in the methods and results across the studies limits the conclusion about which technique is the best, specific radiologic phenotypes may be identified. Qualitative/quantitative MR changes in the substantia nigra do not discriminate between different parkinsonisms. In the absence of extranigral abnormalities, the diagnosis of PD is more probable, whereas basal ganglia changes (mainly in the putamen) suggest the diagnosis of an atypical parkinsonian syndrome. In this context, changes in pons, middle cerebellar peduncles, and cerebellum suggest the diagnosis of MSA, in midbrain and superior cerebellar peduncles the diagnosis of PSP, and in whole cerebral hemispheres (mainly in frontoparietal cortex with asymmetric distribution) the diagnosis of Corticobasal Syndrome.

## 1. Introduction

Neurodegenerative parkinsonian syndromes represent a group of neurological disorders characterized by predominant motor impairment associated with nonmotor symptoms such as cognitive, psychiatric, autonomic, and sleep disorders. Idiopathic Parkinson's disease (PD) underlies the majority of cases followed by the so-called atypical parkinsonian syndromes (APSs), that is, Progressive Supranuclear Palsy (PSP), Multiple System Atrophy (MSA), and Corticobasal Syndrome (CBS). These forms have different clinical features, response to treatment, and prognosis, but clinical overlaps are frequent especially in the early stages of the disease and may lead to misdiagnosis [1]. As the definite diagnosis of these disorders requires a neuropathological confirmation and the accuracy of the clinical diagnosis is suboptimal [1], many studies have searched for different biomarkers able to increase the* in vivo* diagnostic accuracy to discriminate different parkinsonian syndromes.

Many putative biomarkers derived from genetic-epigenetic, neurophysiological, and imaging techniques have been evaluated in order to determine their diagnostic accuracy in discriminating PD from APSs [2]. Brain magnetic resonance (MR) represents one of the best putative sources of biomarkers in this field, because of its relative feasibility, the absence of invasiveness, and the availability in different clinical settings.

First, brain MR imaging is useful to discriminate degenerative parkinsonian syndromes from nondegenerative forms including vascular parkinsonism, basal ganglia or thalamic tumors, normal pressure hydrocephalus, manganism, metabolic disorders such as uremic-diabetic syndrome or extrapontine myelinolysis, and inflammatory diseases [3]. Second, it may show peculiar brain alterations that are typical of different neurodegenerative diseases [3]. Third, the application of advanced neuroimaging techniques, such as morphometric-volumetric analyses, diffusion-weighted imaging (DWI), diffusion tensor imaging (DTI), tractography, magnetization transfer imaging (MTI), proton MR spectroscopy (^1^H-MRS), and iron-content sensitive imaging, may provide macro- and microstructural and biochemical quantitative markers of neurodegeneration* in vivo*. Overall, these techniques may be analysed by either voxel-wise methods that are applied to groups of subjects or regions of interest based methods that may be applied to single subjects and therefore provide information regarding brain regional degeneration. The introduction of high-field scanners (≥1.5 Tesla) has allowed a further improvement of the diagnostic potentiality of MR, not only for research purposes but also in clinical practice, providing a better signal/noise ratio and better spatial resolution in a shorter acquisition time. This is particularly true for the iron-sensitive sequences, such as T2^*∗*^-weighted and susceptibility-weighted images. High magnetic fields surely improved the advanced MR techniques, such as DTI, ^1^H-MRS, and functional MRI. On the other hand, some qualitative MRI markers that are detectable by using lower field scanner may lose value using 3T or higher scanners (see below).

Our aim is to review the diagnostic usefulness of qualitative and quantitative brain MR techniques in the differential diagnosis among degenerative parkinsonian disorders. We have considered only studies that focused on the differential diagnosis between PD and APSs using methods that are readily applicable to individual patients in routine clinical practice. We did not consider the results of functional MRI studies as well as of voxel-wise analyses because of the lack of validation for the single patient use, despite their importance to pave the way to further studies focused on specific targeted regions.

## 2. Qualitative Brain MRI 

**Figure 1:**
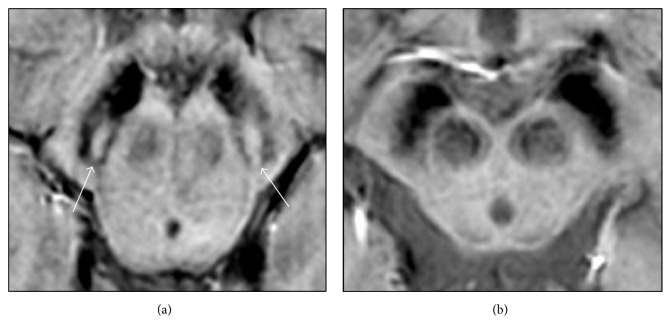
(a) High-resolution MRI (Philips Ingenia 3 T SWIp sequence) in a healthy subject: nigrosome 1 is evident as a linear, comma- or wedge-shaped hyperintense area surrounded by low signal intensity structures of the pars compacta of the substantia nigra, resembling a swallow tail (arrows). (b) In a patient with PD, nigrosome 1 is not visible bilaterally.

Conventional MR techniques are sequences that are routinely used on a standard clinical MR scanner. Overall, these are generally qualitatively evaluated and are mainly represented by T1-weighted, T2-weighted spin-echo (SE), T2-weighted fluid-attenuated inversion-recovery (FLAIR), and proton density images. Furthermore, in recent years, sequences such as diffusion-weighted, T2^*∗*^-weighted, and susceptibility-weighted images are entering into the daily clinical practice in many clinical centres. Conventional brain MRI is frequently performed in the diagnostic pipeline of a patient with parkinsonism, and it does not usually disclose clear abnormalities in PD patients. The substantia nigra (SN) involvement can be indirectly identified as a hypointensity on T2- or T2^*∗*^-weighted images due to secondary iron deposition, but with low sensitivity and specificity. Recent studies using 3 T and 7 T MR scanners and susceptibility-weighted images (SWI) have suggested a new sign of nigral degeneration, the absence of the “swallow tail” sign [4]. This is a dorsolateral hyperintensity within the otherwise hypointense pars compacta and seems to correspond to the anatomic location of nigrosome-1, a small group of dopaminergic cells detectable in healthy individuals but not in PD patients (Figure 1) [5, 6]. However, the absence of this sign has been also reported in APSs other than PD, providing a useful tool to discriminate parkinsonian syndromes from other diseases, for example, essential tremor or psychogenic disorders, but not PD from APSs [7, 8].

Other sequences may also be able to detect nigral neuronal loss in PD patients including a variety of inversion-recovery images [9] and a recently developed neuromelanin-sensitive T1-weighted image [10, 11]. In particular, the latter may become a useful tool in clinical practice as the visual inspection of neuromelanin-sensitive MR images by experienced neuroradiologists provides results comparable to quantitative width measurement in the detection of early stage PD SN changes [11]. As for other SN MR changes, however, neuromelanin-sensitive T1-weighted images do not represent a tool to discriminate among different forms of parkinsonian syndromes.

As concerns APSs, that is, MSA, PSP, and CBS, conventional MRI may disclose specific alterations involving further brain structures in addition to the SN, providing radiological signs useful for the differential diagnosis [3].

**Figure 2:**
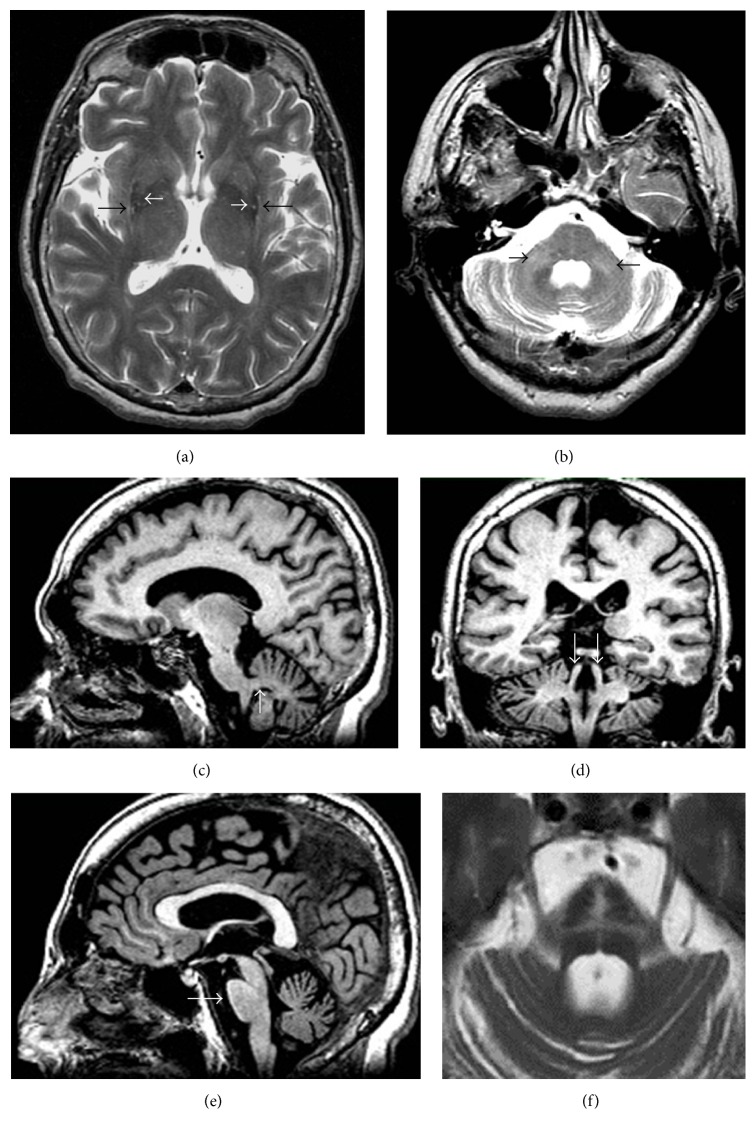
MRI findings in MSA. (a) Bilateral posterior putaminal T2-hypointensity (white arrows) with “slit-like” marginal hyperintensities (black arrows). (b) T2-hyperintensities of middle cerebellar peduncles (black arrows). (c) Atrophy of middle cerebellar peduncles (white arrow) on sagittal 3D T1 image. (d) Normal superior cerebellar peduncles (white arrows) on coronal 3D T1 image. (e) Pons atrophy (white arrow) on sagittal 3D T1 image. (f) Pontine cruciform T2- hyperintensity (“hot-cross bun” sign). In (b, d, e, f), cerebellar atrophy is also visible.

MSA is clinically characterized by extrapyramidal signs accompanied by various degrees of autonomic failure, pyramidal, and cerebellar signs. Two variants have been distinguished, the cerebellar and parkinsonian types (MSA-C and MSA-P, resp.), depending on the prevalence of cerebellar or parkinsonian features. The pathological hallmark is the deposition of *α*-synuclein within oligodendrocytes cytoplasm, leading to different degrees of striatonigral or olivopontocerebellar inclusions and degeneration depending on the disease subtype [12, 13]. The MSA-P may be associated with putaminal atrophy and T2- hypointensity, mainly involving the posterior portion, and a “slit-like” marginal hyperintensity of the putamen at 1.5 T scans (Figure 2(a)). Unfortunately, the hyperintense putaminal rim on the T2-weighted imaging on 3 T scans is a nonspecific finding, as it can be seen also in healthy subjects [14]. On the other hand, a putaminal hypointensity on iron-sensitive images, such as T2^*∗*^-weighted or SWI, detected using 1.5 T scans and even more 3 T scans, helps to discriminate MSA-P from PD and other APSs [15–18]. As disease progresses, several radiological abnormalities of the infratentorial compartment may become apparent, that is, cerebellar atrophy, middle cerebellar peduncle (MCP) atrophy with or without T2 or FLAIR signal increase, pons atrophy, and a pontine cruciform T2 and proton density hyperintensity (“hot-cross bun sign”) [3] (Figures 2(b)–2(f)). These infratentorial abnormalities are more frequent and earlier in MSA-C and can occur in other neurodegenerative diseases such as spinal cerebellar ataxia or fragile-X premutation syndrome [3].

PSP is mainly characterized by axial parkinsonian signs with early postural instability and falls, supranuclear gaze palsy, and poor levodopa response [19]. The neuropathological hallmarks are represented by brain tau-protein deposits, mainly within the basal ganglia and the brainstem, along with different degrees of midbrain, cerebellar, and cortical degeneration [20]. Depending on the phenotypic characteristics of onset and disease progression, PSP spectra may include different disorders such as a classical variant, the so-called Richardson's syndrome (PSP-RS), and the PSP-parkinsonism variant (PSP-P), which are characterized by different clinical features depending on a differential tau-pathology distribution [19, 20].

**Figure 3:**
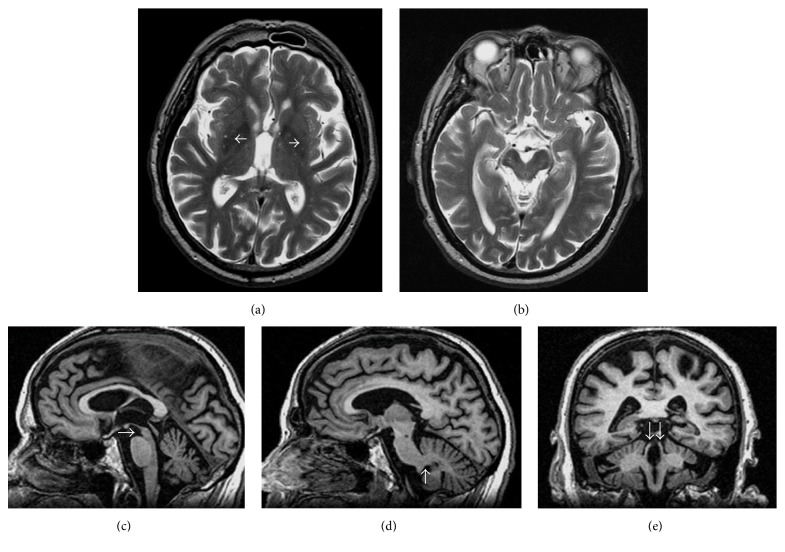
Typical MRI changes disclosed in PSP. (a) Bilateral posterior putaminal T2-hypointensity (white arrows). (b) Atrophy of the midbrain tegmentum on axial T2 image, with thinning of cerebral peduncles resulting in the concavity at the lateral margin of the midbrain, resembling Mickey Mouse or the flower morning glory. (c) Midbrain atrophy (white arrow) with a concave upper profile on sagittal 3D T1 image (brainstem profile resembling a “penguin silhouette” or a “hummingbird”). (d) Normal middle cerebellar peduncles (white arrow) on sagittal 3D T1 image. (e) Atrophy of superior cerebellar peduncles (white arrows) on coronal 3D T1 image.

MRI changes disclosed in PSP patients include atrophy and T2-hypointensity of putamen, mainly in the posterior portion, and of the globus pallidus, midbrain atrophy (“penguin silhouette” or “hummingbird” on sagittal images and “Mickey Mouse appearance” or “morning glory sign” on axial images), superior cerebellar peduncle (SCP) atrophy, third ventricle dilation, and periaqueductal T2-hyperintensities [3, 21] (Figure 3).

**Figure 4:**
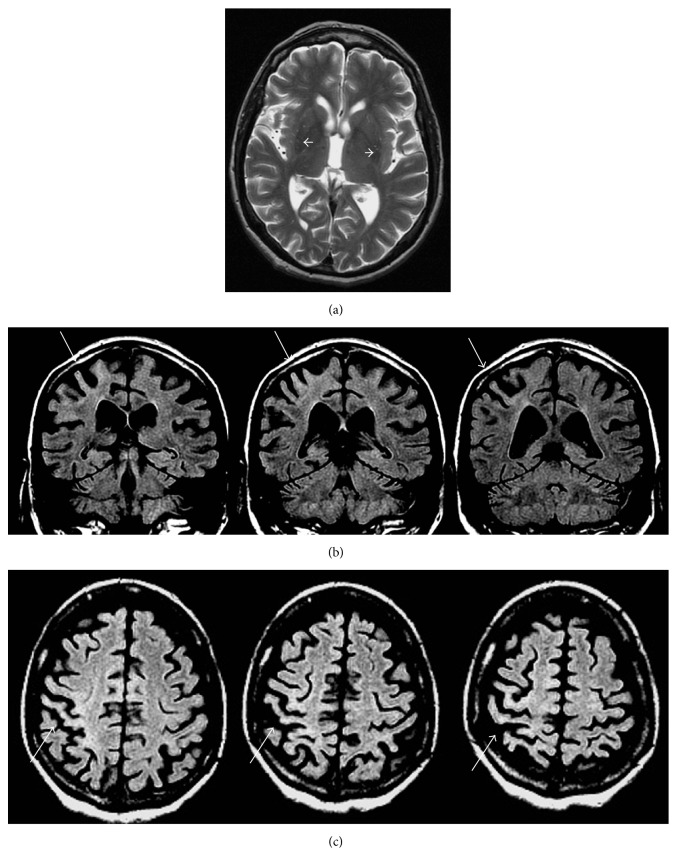
MRI changes disclosed in CBS. (a) Bilateral posterior putaminal T2-hypointensity (white arrows), mainly in the right side, where the putamen appears atrophic. (b)-(c): asymmetric cortical atrophy, mainly at the level of the right primary sensory-motor cortex associated with FLAIR hyperintensity (white arrows), on coronal (b) and axial images (c).

CBS represents a clinical phenotype characterized by an asymmetric, predominantly akinetic-rigid parkinsonism with poor levodopa responsiveness, limb dystonia, and higher cortical impairment represented by limb and buccolingual apraxia, alien limb phenomena, and speech disturbances [22]. Possible underlying pathologies of CBS include corticobasal degeneration, Alzheimer's disease, PSP, frontotemporal lobe degeneration, and prion disease. Putaminal atrophy and T2-hypointensity, with an asymmetric involvement, can be visible in CBS patients. Furthermore, asymmetric cortical atrophy, mainly at the level of the primary sensory-motor cortex and sometimes associated with FLAIR hyperintensity, may occur [3] (Figure 4).

All these MRI markers have good specificity but lack sensitivity, especially in the early stages. Overall, they seem to contribute little over and above the clinically based diagnosis but may be helpful when the clinical diagnosis is uncertain [23]. In particular, the presence of putaminal or infratentorial abnormalities is particularly indicative of APSs.

Finally, other than for APSs, conventional MRI is useful to diagnose other, rarer forms of degenerative parkinsonism with earlier age of onset [3]. Important examples are the marked pallidal hypointensity within a hyperintense central core of necrosis suggesting pantothenate kinase-associated neurodegeneration (“eye of the tiger”) [24], T1 hyperintensities of SN and pallidum suggesting manganese accumulation [25], and T2 and FLAIR hyperintensities at the level of the basal ganglia, thalami, and brainstem (“double panda sign”), seen in Wilson's disease [26, 27].

## 3. Quantitative Brain MR

Advanced brain MR techniques represent a group of sequences and analytic methods that allow a quantitative evaluation of biochemical and macro- and microstructural alterations. The main techniques are represented by ^1^H-MRS, sequences for iron-content detection, high-resolution 3D T1-weighted sequences with morphometric and volumetric analyses, MTI, DWI, DTI, and tractography.

MRS is a noninvasive method that permits the measurement of the concentration of specific biochemical compounds in the brain in precisely defined regions guided by MRI. With MRS, spectra of many biologically important metabolites can be quantified. ^1^H-MRS is the most used in clinical practice and can detect N-acetylaspartate- (NAA-) containing compounds (markers for neuronal integrity, viability, and number), choline-containing compounds (Cho) (major constituents of the membranes), creatine-phosphocreatine (Cr) (whose peak is relatively stable and commonly used as a concentration internal reference), glutamate and glutamine (Glx) (linked to excitatory neurotransmission), myo-inositol (mI) (a glial marker), scyllo-inositol (closely coupled with mI amount), lactate (the end product of anaerobic glycolysis), and lipids [28, 29].

Several MRI techniques have been used to measure nonheme iron-content in brain. The most iron-sensitive and used parameters are T2^*∗*^ or T2′, and T2 to a lesser extent. Relaxometry is frequently used to evaluate the different relaxation rates R2 (1/T2), R2^*∗*^ (1/T2^*∗*^), and R2′(1/T2′ = R2^*∗*^ − R2) [30]. Further techniques include mapping of field dependent transverse relaxation rate increase (FDRI) [31], magnetic field correlation (MFC) [32], phase imaging [33], susceptibility-weighted imaging (SWI) [34], direct saturation imaging [35], and the recently developed quantitative susceptibility mapping (QSM) [36]. All these techniques provide parameters that correlate with iron content and that can be evaluated by either voxel-wise analysis methods or regions of interest based methods. However, T2^*∗*^-weighted sequences and SWI are probably the most feasible in daily clinical practice.

A number of methods are available to measure brain atrophy on high-resolution 3D T1-weighted MRI scans. These include manual morphometric measurements [37], voxel based morphometry (VBM) performing a voxel-by-voxel comparison of the density of brain gray matter and white matter across groups of subjects [38], volumetric and shape analysis of subcortical structures, and estimation of cortical thickness by using different software [39, 40].

DWI is sensitive to the random thermal movement of water molecules (Brownian motion) in neural tissues and is able to identify spatially resolved microstructural brain damage, via the diffusivity values (apparent diffusion coefficient, ADC; mean diffusivity, MD), which are typically elevated in brain areas where neurodegeneration occurs. DTI, based on a greater number of gradient directions and using the tensor model, allows calculating further diffusivity parameters other than MD, such as the degree of anisotropy of such diffusion, represented by fractional anisotropy (FA), axial diffusivity (AD), and radial diffusivity (RD), which are all parameters sensitive to neuronal and/or glial integrity. Overall, these sequences (DWI and DTI) may be analyzed by regions of interest or voxel-wise methods [41–43]. Another way to study DTI data is to reconstruct and evaluate the integrity of WM tracts that physically connect the different regions of the brain, using a number of tractography algorithms based on the anisotropic diffusion of water molecules along the axons [44].

MTI uses an off-resonance radiofrequency pulse to saturate protons in macromolecules and water molecules that are bound to macromolecules. During the pulse sequence, the saturated protons may enter the free pool of protons, primarily water, or may transfer their magnetization to free the surrounding water protons (magnetization transfer ratio, MTR), causing a decrease in the MR signal [45]. Low MTR may be caused by a reduction in the integrity of macromolecular matrix reflecting damage to the neuronal/axonal membrane or to the myelin [45].

Considering the possibility of evaluating neurodegeneration* in vivo*, these sequences have been applied to neurodegenerative disorders for both pathophysiological and diagnostic purposes, providing information regarding the different biochemical and microstructural aspects of neurodegeneration (Table 1).

Because of the possibility to obtain an objective quantification of regional brain alterations, these techniques may be useful in the differential diagnosis, in the follow-up, and in the response to treatment of patients with brain disorders.

The main findings provided by the studies that used quantitative brain MR techniques in patients with parkinsonian syndromes [37, 46–108] are reported in detail in Table 2, divided by the different methods applied and in Table 3 by the different brain structures studied, whereas they are briefly summarized in the following paragraphs according to the different diseases.

### 3.1. PD

The majority of studies that applied advanced MR techniques to PD patients provided an* in vivo* demonstration of neurodegenerative changes, mainly represented by neuronal loss and dysfunction, within the SN, represented by increased iron deposition [109, 110], reduced neuromelanin content [10], NAA concentration [111], and altered DTI metrics [112, 113]. Although these studies provided important information regarding PD pathophysiology and early diagnosis of degenerative parkinsonism, these alterations do not represent accurate markers for the discrimination between PD and APSs, reflecting the comparable SN involvement among the different diseases.

### 3.2. MSA

Since the presence of predominant cerebellar signs and symptoms is uncommon in PD, the differential diagnosis with MSA-C is usually less challenging in the clinical setting. Therefore, the majority of the studies that addressed the differential diagnosis between MSA and other parkinsonisms focused on the MSA-P variant (Table 3). In particular, the main MR features related to MSA-P are represented by putaminal, pontine, MCP, and cerebellar biochemical and structural alterations compared to PD, PSP, and healthy controls (HC) (Tables 2 and 3).

Putaminal alterations indicating neuronal loss, represented by volumetric reduction, increased iron deposition, increased ADC/MD, and reduced NAA, have all been documented as significant differences between MSA-P and PD, PSP, or HC (Tables 2 and 3). In particular, putaminal ADC/rTrace(D)/MD increase and volume reduction may represent accurate markers for the discrimination of MSA-P from PD with moderate-to-high sensitivity and specificity (66.7–100% and 63.6–100%, resp.) [61, 62, 64, 66, 68, 72, 75, 77, 83, 96, 97]. In addition, the increase of iron percentage in the putamen, detected by T2^*∗*^-weighted sequences and SWI, is able to distinguish MSA-P from PD with moderate-to-high accuracy (84–88%) [94, 95]. Similar results have also been reported in the globus pallidus and caudate (Tables 2 and 3), with high specificity (93.7%) but lower sensitivity (62.5–75%) in discriminating MSA-P from PD [66].

Nevertheless, because of the overall lack of specificity of basal ganglia changes, they may be useful in discriminating MSA from PD and HC but not from other APSs.

With regard to the infratentorial compartment, macro- and microstructural changes mainly restricted to the pons and the MCPs, along with pontine biochemical neurodegenerative alterations, have been reported in MSA-P patients compared to PD, PSP, and HC. In particular, it has been demonstrated that a reduction of MCPs width and an increase in its ADC discriminate MSA-P from PD with high sensitivity and specificity (91–100% and 84–100%, resp.) [48, 66, 67, 77]. Also increased ADC in pons and cerebellum have moderate-to-high sensitivity (70% and 60%, resp.) and specificity (70% and 87.5%, resp.) in discriminating MSA-P from PD [75]. FA values in the caudate and RD values in MCPs have also demonstrated 90% sensitivity and 100% specificity in discriminating MSA-P from PSP [82].

In addition, cerebellar structural alterations have been reported in both MSA-P and MSA-C, including NAA reduction on MR spectroscopy (Tables 2 and 3). All these findings have a good diagnostic value allowing the discrimination between MSA and other APSs.

### 3.3. PSP

The majority of studies investigating the usefulness of advanced brain MR techniques in the differential diagnosis of PSP compared to PD, MSA-P, and HC focused on biochemical and macro- and microstructural alterations affecting the lenticular nucleus, midbrain, and superior cerebellar peduncles (Tables 2 and 3).

At a supratentorial level, the main findings are represented by the demonstration of neurodegenerative changes in the putamen and the globus pallidus, characterized by volume loss and neuronal-axonal degeneration, indicated by iron deposition, reduced NAA content, and increased water diffusivity (Tables 2 and 3). Similar results were found in the caudate nucleus and the thalamus (Tables 2 and 3). In particular, a reduction in putaminal and thalamic volumes showed a moderate-to-high accuracy (83%), sensitivity (93% and 73%, resp.), and specificity (90% and 70%, resp.) in discriminating PSP-RS from PD [55]; pallidal volume also demonstrated a moderate-to-high accuracy (86%) [55]. In addition, increased putaminal ADC showed moderate-to-high sensitivity (75–90%) and specificity (77–100%) in discriminating PSP from PD [62, 66, 70]. Quantitative markers derived from iron-sensitive sequences demonstrated high accuracy in discriminating PSP from PD: in particular, phase shift values in the thalamus and in the pallidus (87%) [95].

As for MSA, basal ganglia changes can discriminate PSP from PD and HC but not from other APSs. Besides the basal ganglia, diffuse structural and biochemical alterations have been demonstrated in PSP compared to PD and HC, in particular within frontal lobes, hippocampus, corpus callosum, and cingulate gyrus (Tables 2 and 3). In addition, corticospinal tract (CST) and dentatorubrothalamic tract (DRTT) microstructural alterations have been reported in PSP patients compared to PD and to PD, MSA-P, and HC, respectively [86, 88]. However, the diagnostic utility of all these abnormalities is still uncertain. Alterations in infratentorial structures have also been demonstrated* in vivo* in PSP when compared to PD, MSA subtypes, and HC. In particular, atrophy and DWI/DTI metrics changes, such as increased ADC and MD and reduced FA, in midbrain and SCPs, along with alterations in the composed morphometric parameters, such as MR parkinsonism index (MRPI), midbrain/pons ratio, and MCP/SCP ratio, seem to be the markers with the best accuracy in discriminating PSP from other parkinsonisms (Tables 2 and 3). Overall, these markers showed a higher accuracy in discriminating PSP from other diseases compared to markers derived from supratentorial structures. In particular, the most accurate markers are represented by midbrain area (95–100 sensitivity and 91.3–98% specificity) [47, 49, 54], SCP diameter (65.5% sensitivity and 93.5% specificity) [49], MCP/SCP ratio (78.8% sensitivity and 88.9% specificity) [37], pons/midbrain ratio (90–96% sensitivity and 90–96% specificity) [37, 51, 54], midbrain/pons ratio (63.6–100% sensitivity and 92.1% specificity) [47, 50], and MRPI (81.8–100% sensitivity and 80.2–100% specificity) [37, 50–52, 54]. Increased MD in SCPs and in the posterior fossa showed moderate accuracy in discriminating PSP-RS from PD (83%) [54].

### 3.4. CBS

Studies focusing on the basal ganglia found biochemical (reduced NAA content [101, 103]) and microstructural (increased diffusivity [70]) putaminal changes in CBS, which may be helpful in discriminating it from PD and HC but not from other APSs. Volume loss in the thalamus was found in CBS* versus* PD and HC, but similar to PSP [84], whereas increased diffusivity in different thalamic regions seems to discriminate CBS from PSP [78].

Taking into account that cortical impairment represents a hallmark of CBS, most of the studies focused on the application of advanced MR techniques to the supratentorial compartment, providing findings that are more helpful. In particular, asymmetric atrophy in frontal, parietal, and occipital lobes indicated by a volumetric study [57] along with microstructural alterations, represented by increased diffusivity in the whole cerebral hemispheres, asymmetrically [70] or in the precentral and postcentral gyri [88], have been reported in CBS patients compared to PD, PSP, and HC. In addition, reduced frontoparietal cortex NAA content [101, 103] and morphometric [57] and DTI changes [80] of the corpus callosum have also been demonstrated in CBS compared to PD and HC, but with few data about the discriminating value compared to the other APSs.

## 4. Discussion 

Currently, a plethora of MR studies focusing on diagnostic markers in parkinsonian syndromes is available in the literature. Earlier studies have highlighted the qualitative MRI changes found in the different atypical parkinsonian syndromes that reflect the underlying neuropathological pattern; these changes showed good specificity but low sensitivity. More recently, several quantitative methods have been introduced in the attempt to increase the sensitivity of MR findings. Despite their promising results, however, the variability of the methods used is still a limitation to their widespread application. Moreover, there is a wide variability of results across studies using the same methodology, which does not allow drawing firm conclusions on the applicability of most of the methods used.

Although structural and biochemical alterations in different brain regions have been reported to have a high diagnostic accuracy, studies evaluating these different features in the same population are few [53, 54, 83, 84, 88, 96]. Therefore, a comparison of the diagnostic properties of different MR markers is difficult. Furthermore, the potential and the results of each technique depend on magnetic fields, resolution, type of pre- and postprocessing, and statistical analysis adopted, possibly affecting the reproducibility of the findings. The image-processing pipeline is crucial to obtain reliable data and can be very long and full of pitfalls (e.g., as regards DTI, susceptibility correction, eddy current correction, head motion correction, estimate of the tensor in each voxel, and extraction of quantitative parameters) [114], and each step is susceptible to sources of bias, which may not only limit the accuracy and precision but can lead to a misinterpretation of the results. Other than technical considerations, a limitation of most of the studies analyzing manually segmented structures is the operator-dependence. Different MRI studies using the same markers have demonstrated a highly variable diagnostic accuracy, with sensitivity and specificity ranging from 100% to much lower values (Table 2). Among all the techniques used in recent years, the simplest and most feasible in clinical practice seems to be the morphometric evaluation of midbrain, pons, cerebellar peduncles, and derived ratios, which has provided the best diagnostic accuracy values [37, 47–54]. However, a multimodal approach including parameters derived from different techniques may increase the global diagnostic accuracy [53].

A further important consideration is that in the vast majority of studies MR findings are compared between clinical, and not pathological, diagnoses, which may also be inaccurate [1]. Furthermore, most of the studies are on patients with long disease duration and it is uncertain whether the discriminating MRI findings would help the diagnosis in the early stage of the diseases.

**Figure 5:**
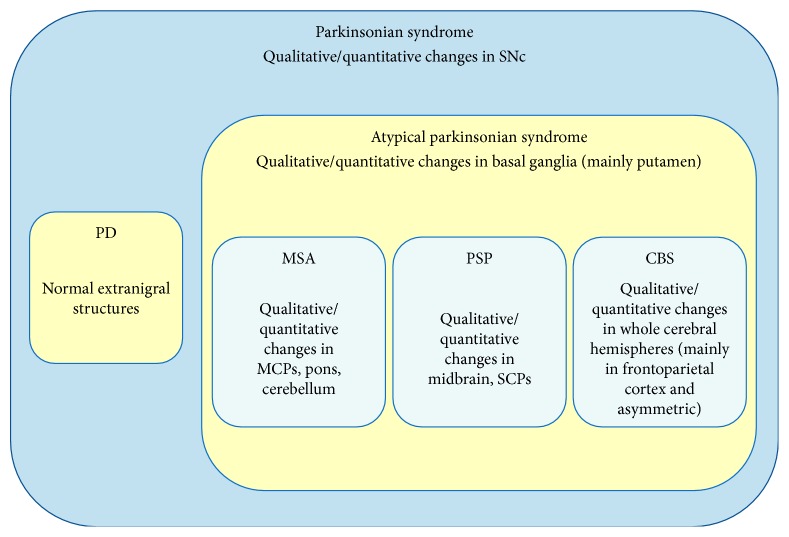
Flowchart of MR diagnosis in parkinsonian syndromes. SNC: substantia nigra pars compacta; PD: Parkinson's disease; MSA: Multiple System Atrophy; PSP: Progressive Supranuclear Palsy; CBS: Corticobasal Syndrome; MCPs: middle cerebellar peduncles; SCPs: superior cerebellar peduncles.

These limitations notwithstanding, the* in vivo* identification of brain pathological patterns more frequently associated with different diseases (Table 3) is undoubtedly useful to better define a specific clinicoradiologic phenotype, which can increase the diagnostic probability of PD or MSA or PSP or CBS. Indeed, MR findings, by a qualitative or quantitative evaluation, can be useful mainly in those cases with an uncertain clinical phenotype. Specifically, qualitative/quantitative changes in the SN pars compacta indicate a degenerative parkinsonism. In this context, we propose the following conclusive considerations: (i) if no extranigral abnormalities are evident, the likelihood of PD diagnosis increases; (ii) if qualitative/quantitative changes are disclosed in the basal ganglia (mainly in the putamen), the likelihood of APS diagnosis increases; (iii) if qualitative/quantitative changes are disclosed in pons, MCPs, and cerebellum, the likelihood of MSA diagnosis increases; (iv) if qualitative/quantitative changes are disclosed in midbrain and SCPs, the likelihood of PSP diagnosis increases; (v) if qualitative/quantitative changes are disclosed in whole cerebral hemispheres (mainly in frontal and parietal cortices with asymmetric distribution), the likelihood of CBS diagnosis increases (Figure 5).

Taking into account the scientific literature, the feasibility in a clinical setting, and the availability of high-field MR scanners, a magnetic field of at least 1.5 Tesla is required by most of the reported advanced techniques. Moreover, although a consensus on the type of sequences needed to discriminate PD from other mimics is lacking, an optimal protocol should include volumetric T1-weighted, axial T2-weighted fast spin-echo/FLAIR, a diffusion-weighted sequence (DWI or better DTI), an iron-sensitive sequence, as T2^*∗*^-weighted or SWI, and ^1^H-MRS acquisition with a single-voxel localized within the basal ganglia and posterior fossa structures, that is, the pons or the cerebellar hemisphere.

In conclusion, MR markers are feasible and useful to increase the diagnostic accuracy of degenerative parkinsonian syndromes, although a rate of misdiagnosis remains currently inevitable. This emphasizes the need for specific biomarkers for the pathology underlying the different parkinsonian syndromes, for example, *α*-synuclein or tau deposits, which along with a well-defined clinicoradiological phenotype can allow a more certain diagnosis. Such biomarkers can be biological, for example, from blood, liquor, skin, submandibular gland, or gut, but also imaging-based. Imaging biomarkers are currently obtained by the use of nuclear medicine tracers, such as amyloid or tau PET ligands, but in the future, similar tracers could be also available for MRI, for example, using nanoparticle-based contrast agents.

## Figures and Tables

**Table 1:** Surrogate quantitative MR markers indicating different features of neurodegeneration. The main markers corresponding to the underlying pathology and the sequences needed are indicated.

	Microstructure-biochemical profile		Macrostructure	
	Marker	Sequence	Marker	Sequence
*Neuronal/axonal loss: degeneration*	ADC, rTrace(D), MD, AD	DW Images	T2 signal intensity	T2-w sequences
	NAA	^1^H-MRS	Area, diameter, volume	Volumetric T1 sequences
	MTR	MTI (T1 or PD-w sequence with off resonance saturation)		

*Glial reaction: gliosis*	mI	^1^H-MRS		

*Myelin disruption*	FA, RD	DW Images	T2 signal intensity	T2-w sequences
	Cho	^1^H-MRS	Volume	Volumetric T1 sequences
	MTR	MTI (T1 or PD-w sequence with off resonance saturation)		

*Iron content*	T2^*∗*^/R2^*∗*^ values	T2^*∗*^/R2^*∗*^-w sequence		
	Phase shift values	SWI phase images		

ADC: apparent diffusion coefficient; MD: mean diffusivity; AD: axial diffusivity; DW: diffusion weighted; NAA: N-acetyl-aspartate; ^1^H-MRS: proton-MR spectroscopy; MTR: magnetization transfer ratio; MTI: magnetization transfer imaging; PD: proton density; mI: myo-inositol; FA: fractional anisotropy; RD: radial diffusivity; Cho: choline; SWI: susceptibility-weighted imaging.

**Table 2:** Studies evaluating quantitative advanced brain MR parameters for the differential diagnosis between Parkinson's disease and atypical parkinsonisms.

Author, year	Magnetic field	Technique	Cohort	Results	Acc/Se/Sp
Kato et al., 2003 [46]	1.5 T	Morphometric measurements	PSP = 8; PD = 12; HC = 10 DD (y, M ± SD): PSP = 6.4 ± 6.2; PD = 6.9 ± 7.2	↓ area of midbrain tegmentum, inferior colliculus, and pontine tegmentum in PSP versus PD	Not reported

Oba et al., 2005 [47]	1.5 T	Morphometric measurements	PSP = 21; MSA-P = 25; PD = 23; HC = 31 DD (y, M ± SD): PSP = 2.8 ± 1.3; MSA-P = 7.8 ± 3.8; PD = 6.6 ± 1.9	↓ midbrain area in PSP versus PD and MSA-P ↓ pons area in MSA-P versus PD and PSP ↓ midbrain/pons ratio in PSP versus PD and MSA-P	Se/Sp for PSP diagnosis: midbrain area = 100%/91.3%; midbrain/pons ratio = 100%/100%

Nicoletti et al., 2006 [48]	1.5 T	Morphometric measurements	MSA-P = 16; PD = 26; HC = 14 DD (y, M ± SD): MSA-P = 5.06 ± 3.38; PD = 5.92 ± 6.46	↓ MCP width in MSA-P versus PD	Se/Sp of MCP width for MSA-P diagnosis = 100%/100%

Quattrone et al., 2008 [37]	1.5 T	Morphometric measurements	PSP = 33; MSA-P = 19; PD = 108; HC = 50 DD (y, M ± SD): PSP = 3.0 ± 1.6; MSA-P = 4.6 ± 3.1; PD = 5.5 ± 4.3	↓ midbrain area in PSP versus PD and MSA-P ↓ pons area in MSA-P versus PD and PSP ↓ SCP width in PSP versus PD and MSA-P ↓ MCP width in MSA-P versus PD and PSP ↑ pons/midbrain ratio in PSP versus PD and MSA-P ↑ MCP/SCP ratio in PSP versus PD and MSA-P ↑ MRPI in PSP versus PD and MSA-P	Se/Sp for PSP diagnosis: pons/midbrain ratio = 90.9%/93.5% (versus PD), 97%/94.7% (versus MSA-P); MCP/SCP ratio = 78.8%/88.9% (versus PD), 93.9%/89.5% (versus MSA-P); MRPI = 100%/100% (versus PD and MSA-P)

Gama et al., 2010 [49]	1.5 T	Morphometric measurements	PD = 21; MSA-C = 11; MSA-P = 8; PSP = 20 DD (y, M ± SD): PD = 6.0 ± 3.66; MSA-C = 3.9 ± 1.62; MSA-P = 5.0 ± 3.2; PSP = 5.6 ± 2.28	↓ midbrain area in PSP and MSA-P versus PD, of PSP versus MSA-C and MSA-P and of MSA-P versus MSA-C ↓ pons area PSP, MSA-P, and MSA-C versus PD, of MSA-C and MSA-P versus PSP ↓ MCP diameter in PSP, MSA-C, and MSA-P versus PD, and MSA-C versus PSP ↓ SCP diameter in PSP and MSA-C vs PD, PSP vs MSA-C and MSA-P	Acc/Se/Sp of midbrain area in discriminating PD, PSP, and MSA-C: 80.0%/65.5%/93.5%, 96.7%/95.0%/97.5%, and 51.7%/66.7%/93.8%, respectively Acc/Se/Sp of pons area in discriminating PD and MSA-C: 90.0%/80.0%/97.1%, 88.3%/77.5%/100% Acc/Se/Sp of MCP diameter in discriminating PD, PSP, and MSA-C: 85.0%/71.4%/96.9% and 90.0%/66.7%/97.8% Acc/Se/Sp of SCP diameter in discriminating PD and PSP: 80.0%/65.5%/93.5%

Hussl et al., 2010 [50]	1.5 T	Morphometric measurements	PSP = 22; MSA-P = 26; PD = 75 DD (y, M ± SD): PSP = 2.88 ± 1.94; MSA-P = 4.09 ± 1.79; PD = 7.49 ± 6.89	↓ midbrain/pons ratio in PSP versus non-PSP ↑ MRPI in PSP versus non-PSP	Se/Sp for PSP diagnosis (versus non-PSP): midbrain/pons ratio = 63.6%/92.1%; MRPI = 81.8%/80.2%

Longoni et al., 2011 [51]	1.5 T	Morphometric measurements	PSP-RS = 10; PSP-P = 10; PD = 25; HC = 24 DD [y, M, SD (range)]: PSP-RS = 3.8 (2.5–7); PSP-P = 5.1 (3–10); PD = 4.9 (1–19)	↑ pons/midbrain ratio in PSP-RS and PSP-P versus PD ↑ MR parkinsonism index in PSP-RS and PSP versus PD	Se/Sp for PSP diagnosis: pons/midbrain ratio = 90%/96% (PSP-RS versus PD) and 60%/96% (PSP-P versus PD); MRPI = 100%/92% (PSP-RS versus PD) and 70%/68% (PSP-P versus PD)

Morelli et al., 2011 [52]	1.5 T	Morphometric measurements	Probable PD = 170; possible PD = 132; PSP = 42; HC = 38 DD [y, M, SD (range)]: probable PD = 7.06 ± 3.9 (3–22); possible PD = 3.06 ± 2.4 (1–10); PSP = 3.57 ± 2.4 (1–15)	↓ SCP diameter, MCP diameter, midbrain area, pons area, midbrain/pons ratio, and ↑ MRPI in PSP versus PD	Acc/Se/Sp of MRPI in discriminating PSP from possible PD: 99.4%/100%/99.2% Acc/Se/Sp of MRPI in discriminating PSP from probable PD: 99.5%/100%/99.4%

Nair et al., 2013 [53]	3 T	Morphometric measurements: volumetry-DTI	PD = 26; MSA = 13 DD (m, M ± SD): PD = 8.9 ± 6.5 (range 1–25); MSA = 3.7 ± 2.1 (range 1–7)	↓ mean width and FA of MCP, anteroposterior diameter and volume of pons, FA and volume of cerebellum, volume of putamen, FA of rostral substantia nigra, and ↑ MD of MCP in MSA versus PD	Overall performance of the decision tree (including mean MCP width and FA and anteroposterior diameter of pons) was 92% sensitivity, 96% specificity, 92% PPV, and 96% NPV

Zanigni et al., 2016 [54]	1.5 T	Morphometric measurements: volumetry-DTI	PSP-RS = 23; PD = 42 DD (y, M ± SD): PSP-RS = 4.2 ± 2.7; PD = 4.0 ± 3.3	↓ MCP and SCP diameters, pons and midbrain areas and ↑ MCP/SCP diameter, pons/midbrain area, and MRPI in PSP-RS versus PD ↑ MD in SCP, thalamus, putamen, pallidus, parietooccipital and prefrontal WM, brain hemispheres, posterior fossa, brainstem, and cerebellar hemispheres in PSP-RS versus PD ↓ FA in SCP, midbrain (SCPs decussation), parietooccipital and prefrontal WM, brain hemispheres, posterior fossa, and brainstem in PSP-RS versus PD ↓ volume of brainstem, nucleus accumbens, globus pallidus, putamina, thalami, and ↑ volume of lateral ventricles in PSP-RS versus PD	Acc/Se/Sp of midbrain area in discriminating PSP-RS from PD: 99%/96%/98% Acc/Se/Sp of pons/midbrain area in discriminating PSP-RS from PD: 97%/96%/90% Acc/Se/Sp of MRPI in discriminating PSP-RS from PD: 95%/87%/93% Acc/Se/Sp of posterior fossa MD in discriminating PSP-RS from PD: 90%/80%/83% Acc/Se/Sp of SCP MD in discriminating PSP-RS from PD: 88%/70%/98% Acc/Se/Sp of thalamic volume in discriminating PSP-RS from PD: 83%/73%/90% Acc/Se/Sp of putaminal volume in discriminating PSP-RS from PD: 83%/93%/70%

Schulz et al., 1999 [55]	1.5 T	Volumetric measurements	PSP = 6; MSA-P = 12; MSA-C = 17; PD = 11; HC = 16 (age matched) DD (y, M ± SEM): PSP = 2.3 ± 0.95; MSA-P 3.42 ± 0.67; MSA-C 3.41 ± 0.57; PD = 6.82 ± 0.7	↓ TIV-corrected brainstem volume in PSP, MSA-P, and MSA-C versus HC ↓ TIV-corrected cerebellar volume in MSA-P and MSA-C versus HC ↓ TIV-corrected caudate volume in PSP and MSA-P versus PD and HC ↓ TIV-corrected putaminal volume in PSP and MSA-P versus PD and HC	Not reported

Cordato et al., 2002 [56]	1.5 T	Volumetric measurements	PSP = 21; PD = 17; HC = 23 DD (m, M ± SD): PSP = 47.7 ± 34.0; PD = 94.3 ± 35.5	↑ ventricular, ↓ whole brain, and frontal GM volumes in PSP versus PD and HC	Se/Sp discriminant function including frontal GM volume in differentiating PSP from PD and HC: 95.2% and 90.9%

Gröschel et al., 2004 [57]	1.5 T	Volumetric measurements	PSP = 33; CBS = 18; HC = 22 DD (y, M ± SD): PSP = 3.6 ± 2.2; CBS 4.6 ± 1.6	↓ brainstem volume (>midbrain) in PSP versus CBS and HC ↓ parietal and occipital lobes volumes (>white matter) in CBS versus PSP and HC ↓ area of CC in CBS versus PSP and HC	Discriminating capacity of a function including midbrain, parietal WM, temporal GM, brainstem, frontal WM, and pons volumes in differentiating PSP from CBS from HC: 84% (95%, 76%, and 83%, resp.)

Paviour et al., 2005 [58]	1.5 T	Volumetric measurements	PSP = 19; MSA = 10; PD = 12; HC = 12 DD (y, M ± SD): PSP = 4.6 ± 1.6; MSA = 5.4 ± 1.7; PD = 13.25 ± 6.7	↓ TIV-corrected SCP volume in PSP versus MSA, PD, and HC	Se/Sp for PSP diagnosis: 74% and 77%

Paviour et al., 2006 [59]	1.5 T	Volumetric measurements	PSP = 18; MSA = 9; PD = 9; HC = 18 DD (y, M ± SD): PSP = 4.6 ± 1.6; MSA = 5.4 ± 1.7; PD = 12.9 ± 4.3	↓ midbrain and SCP volumes in PSP versus MSA-P, PD, and HC ↓ frontal lobe volume in PSP versus PD and HC ↑ 3rd ventricle volume in PSP versus HC ↓ cerebellar and pontine volumes in MSA-P versus PD and HC ↓ midbrain volume in MSA-P versus HC	Se/Sp for regression analysis including midbrain, SCPs, frontal lobe, 3rd ventricle, and whole brain volumes in discriminating PSP from MSA-P, PD, and NC: 88.9% and 97.3% Se/Sp of midbrain, SCP, pons, and cerebellar volumes in discriminating PSP from MSA-P: 94.4% and 88.9%

Messina et al., 2011 [60]	1.5 T	Volumetric measurements	PSP = 32; MSA-P = 15; PD = 72; HC = 46 DD (y, M ± SD): PSP = 3.53 ± 3.5; MSA-P = 3.07 ± 2.2; PD = 6.19 ± 4.5	↓ cerebellar cortex, thalamus, putamen, pallidum, hippocampus, and brainstem and ↑ lateral, 3rd and 4th ventricles volumes in PSP versus PD and HC ↓cerebellar cortex, putamen, pallidum, hippocampus, and brainstem and ↑ 4th ventricle volumes in MSA-P versus PD and HC ↓thalamus volume in PSP versus MSA-P	Not reported

Schocke et al., 2002 [61]	1.5 T	DWI	MSA-P = 10; PD = 11; HC = 7 DD (y, M ± SD): MSA-P = 2.8 ± 0.9; PD = 2.9 ± 1.1	↑ rADC in putamen in MSA-P versus PD	Se/Sp for MSA-P diagnosis: 100%/100%

Seppi et al., 2003 [62]	1.5 T	DWI	PSP = 10; MSA-P = 12; PD = 13 DD (y, M ± SD): PSP = 2.7 ± 1.1; MSA-P = 2.9 ± 1.1; PD = 3.0 ± 1.2	↑ rADC in putamen, caudate, and pallidum in PSP versus PD ↑ rADC in putamen, caudate in MSA-P versus PD	Se/Sp for MSA-P diagnosis (versus PD): putaminal rADC = 100%/100%. Se/Sp for PSP diagnosis (versus PD): putaminal rADC = 90%/100%. No discrimination between MSA-P and PSP

Schocke et al., 2004 [63]	1.5 T	DWI	MSA-P = 11; PD = 17; HC = 10 DD (y, M ± SD): MSA-P = 3.9 ± 1.9; PD = 3.7 ± 1.8	↑ rTrace(D) in putamen, caudate, and pallidum in MSA-P versus PD	Se/Sp not reported (but no overlap between MSA-P and PD using putaminal rTrace(D))

Seppi et al., 2004 [64]	1.5 T	DWI	MSA-P = 15; PD = 17; HC = 10 DD (y, M ± SD): MSA-P = 3.1 ± 1.5; PD = 3.9 ± 0.9	↑ rADC in putamen in MSA-P versus PD	Se/Sp for MSA-P diagnosis: 93%/100% (higher compared with [^123^I]IBZM-SPECT)

Seppi et al., 2006 [65]	1.5 T	DWI	MSA-P = 15; PD = 20; HC = 11 DD (y, M ± SD): MSA-P = 3.5 ± 2.1; PD = 3.9 ± 1.8	↑ rTrace(D) in putamen in MSA-P versus PD	Se/Sp not reported [but no overlap between MSA-P and PD using posterior putaminal rTrace(D)]

Nicoletti et al., 2006 [66]	1.5 T	DWI	PSP = 16; MSA-P = 16; PD = 16; HC = 15 DD (y, M ± SD): PSP = 3.3 ± 2.5; MSA-P = 4.9 ± 4.0; PD = 7.5 ± 5.8	↑ rADC in putamen, caudate, pallidum, thalamus, MCP, and pons in MSA-P versus PD ↑ rADC in putamen, caudate, pallidum, and thalamus in PSP versus PD ↑ rADC in putamen, MCP in MSA-P versus PSP	Se/Sp for MSA-P diagnosis: MCP rADC = 100%/100% (versus PD and versus PSP); putaminal rADC = 100%/100% (versus PD), 100%/81.2% (versus PSP); pallidal rADC = 62.5%/93.7% (versus PD); caudate rADC = 75%/93.7% (versus PD). Se/Sp for PSP diagnosis (versus PD): putaminal rADC = 75%/100%

Paviour et al., 2007 [67]	1.5 T	DWI	PSP = 20; MSA-P = 11; PD = 12; HC = 7 DD (y, M ± SD): PSP = 4.5 ± 1.8; MSA = 5.4 ± 1.6; PD = 13.3 ± 6.7	↑ rADC in MCP and rostral pons in MSA-P versus PSP and PD	Se/Sp for MSA-P diagnosis (versus PSP and versus PD): MCP rADC = 91%/84%

Köllensperger et al., 2007 [68]	n.a.	DWI	MSA-P = 9; PD = 9; HC = 9 DD (y, M ± SD): MSA = 6.4 ± 2.35; PD = 11.3 ± 6.07	↑ rTrace(D) in putamen in MSA-P versus PD	Se/Sp for MSA-P diagnosis: 100%/100% (higher compared with tilt test and heart MIBG scintigraphy)

Nicoletti et al., 2008 [69]	1.5 T	DWI	PSP = 28; MSA-P = 15; PD = 15; HC = 16 DD (y, M ± SD): PSP = 3.2 ± 1.7; MSA = 5.1 ± 4.0; PD = 8.6 ± 3.6	↑ rADC in SCP in PSP versus PD and versus MSA-P	Se/Sp for PSP diagnosis: 100%/100% (versus PD), 96.4%/93.3% (versus MSA-P). No basal ganglia analysis

Rizzo et al., 2008 [70]	1.5 T	DWI	PSP-RS = 10; CBS = 7; PD = 13; HC = 9 DD (y, M ± SD): PSP-RS = 4 ± 3; CBS = 4 ± 3; PD = 14 ± 8	↑ ADC_ave_ in putamen and SCP in PSP-RS versus PD ↑ ADC_ave_ in putamen in CBS versus PD ↑ hemispheric median ADC_ave_ in CBS versus PD and PSP-RS ↓ HSR in CBS versus PD and PSP-RS	Se/Sp for PSP-RS diagnosis (versus PD): putaminal ADC_ave_ = 80%/77%; SCP ADC_ave_ = 90%/85%. Se/Sp for CBS diagnosis: putaminal ADC_ave_ = 86%/92% (versus PD); hemispheric median ADC_ave_ = 86%/85% (versus PD), 100%/90% (versus PSP-RS); HSR = 100%/100% (versus PD and PSP-RS)

Pellecchia et al., 2009 [71]	1.5 T	DWI	MSA-P = 9; MSA-C = 12; HC = 11 DD (y, M ± SD): MSA-P = 4.1 ± 1.4; MSA-C = 4.1 ± 2.2	↑ trace (D) values in whole and anterior putamen in MSA-P versus MSA-C and HC ↑ trace (D) values in the cerebellum (WM) and MCP in MSA-C versus MSA-P and HC	

Umemura et al., 2013 [72]	1.5 T	DWI (and ^123^I-metaiodobenzylguanidine (MIBG) cardiac scintigram)	PD = 118; MSA-P = 20 DD (y, M ± SD): PD = 6.8 ± 4.9; MSA-P = 3.6 ± 1.8	↑ putaminal ADC in MSA-P versus PD	Se/Sp for MSA-P diagnosis by ADC test: 85.0% and 89.0%

Tsukamoto et al., 2012 [73]	3 T	DWI	PD = 17; PSP = 20; MSA = 25 (5 MSA-P; 20 MSA-C); HC = 18 DD (y, M ± SD): PD = 6.0 ± 3.0; PSP = 4.0 ± 3.0; MSA = 3.4 ± 2.6	↑ rADC in midbrain and globus pallidus in PSP versus PD, MSA, and HC ↑ rADC in the SCP and in the head of the caudate nucleus in PSP versus MSA and HC ↑ rADC in pons, middle cerebellar peduncle, cerebellar white matter, and dentate nucleus in MSA versus PD, PSP, and HC ↑ rADC in the posterior putamen in MSA versus PSP ↑ rADC values in the putamen, globus pallidus, and head of caudate nucleus in MSA-P versus MSA-C ↑ rADC in the pons, middle cerebellar peduncle, and cerebellar white matter in MSA-C versus MSA-P	Not reported

Blain et al., 2006 [74]	1.5 T	DTI	PSP = 17; MSA = 17 (10 MSA-P and 7 MSA-C); PD = 12; HC = 12 DD (y, M ± SD): PSP = 5.3 ± 2.4; MSA = 5.0 ± 2.3; PD = 6.9 ± 2.0	↑ MD in MCP and pons in MSA versus PSP and PD ↓ FA in MCP in MSA versus PSP and PD ↑ MD in decussation of SCP in PSP versus MSA and PD ↓ FA in decussation of SCP in PSP versus PD	Se/Sp not reported

Ito et al., 2007 [75]	3 T	DTI	MSA = 20 (10 MSA-P and 10 MSA-C); PD = 21; HC = 20 DD (y, M ± SD): MSA = 4 ± 2; PD = 10 ± 8	↑ ADC in putamen, pons, and cerebellum in MSA-P versus PD ↓ FA in putamen, pons, and cerebellum in MSA-P versus PD	Se/Sp for MSA-P diagnosis (versus PD): ADC: putamen = 70%/63.6%, pons = 70%/70%, cerebellum = 60%/87.5%; FA: putamen = 70%/87.5%, pons = 70%/100%, and cerebellum = 70%/63.6% 90% of MSA-P had ↑ ADC and ↓ FA in any of the three areas

Ito et al., 2008 [76]	1.5 T	DTI/DWI	PSP = 7; PD = 29; HC = 19 DD (y, M ± SD): PSP = 3.5 ± 1.6; PD = 4.8 ± 3.3	↑ ADC in CC1 in PSP versus PD and HC and in CC2 in PSP versus PD ↓ FA in CC1 in PSP versus HC and in CC2 in PSP versus PD and HC	Se/Sp of ADC in CC1 and CC2 in differentiating PSP from PD: 100% and 75.9%, and 49.2% and 82.8%, respectively Se/Sp of FA in CC1 and CC2 in differentiating PSP from PD: 85.7% and 65.5%, and 28.5% and 90.0%, respectively

Chung et al., 2009 [77]	1.5 T	DWI	PD = 12; MSA-P = 10; HC = 10 DD (m, M ± SD): PD = 30.4 ± 22.03; MSA-P = 23.6 ± 12.82	↑ rADC in dorsal putamen and MCP in MSA-P versus PD and HC	Se/Sp rADC in dorsal putamen in differentiating MSA-P from PD: 66.67%/80% Se/Sp rADC in MCP in differentiating MSA-P from PD: 91.67%/100%

Erbetta et al., 2009 [78]	1.5 T	DTI/DWI	PSP = 9; CBS = 11; HC = 7 DD (y, M ± SD): PSP = 3.8 ± 2.2; CBS = 3.0 ± 1.1	↑ ADC in anterior, medial thalamus, and SCP and ↓ FA in anterior cingulum in PSP versus CBS ↑ ADC in precentral and postcentral gyri in CBS versus PSP	Not reported

Focke et al., 2011 [79]	3 T	DTI	PSP = 9; MSA-P = 10; PD = 12; HC = 13 DD (y, M ± SD): PSP = 2.5 ± 2.3; MSA = 4.5 ± 2.7; PD = 5.8 ± 3.8	↑ MD in pallidum and SN in PSP versus PD and in SN in PSP versus MSA-P	Se/Sp not reported. No differences in other basal ganglia and in other groups of patients

Boelmans et al., 2010 [80]	1.5 T	DTI	CBS = 14; PD = 14; HC = 14 DD (m, M ± SD): CBS = 40.9 ± 18.9; PD = 34.8 ± 18.7	↑ MD in CC in CBS versus PD and HC ↓ FA in middle-dorsal (sensory) CC in CBS versus PD and HC	Se/Sp MD in the whole CC in differentiating CBS from PD: 79% both Se/Sp MD in the posterior truncus CC in differentiating CBS from PD: 86% and 71%

Nicoletti et al., 2013 [81]	1.5 T	DTI	PD = 10; MSA-P = 9; MSA-C = 7; PSP-RS = 17; HC = 10 DD (y, M ± SD): PD = 8.3 ± 2.5; MSA-P = 4.4 ± 1.2; MSA-C = 5.4 ± 1.1; PSP-RS = 5.9 ± 1.8	↑ MD infratentorial compartment, brainstem, cerebellar vermis, and hemispheres in MSA-P and MSA-C versus PD and HC ↑ MD infratentorial compartment, brainstem, and cerebellar hemispheres in MSA-P and MSA-C versus PSP-RS ↑ MD infratentorial compartment, brainstem, and cerebellar vermis in PSP-RS versus PD and HC ↑ MD in cerebellar vermis in MSA-C versus MSA-P	Se/Sp of MD of cerebellar hemispheres in discriminating MSA from other groups: 100%/100%

Prodoehl et al., 2013 [82]	3 T	DTI	PD = 15; MSA-P = 14; PSP = 12; ET = 14; HC = 17 DD (y, M ± SD): PD = 10.5 ± 7.3; MSA-P = 7.4 ± 4.0; PSP = 10.5 ± 2.5; ET = 28.2 ± 21.0	FA of SN, AD of putamen, and MD of dentate in discriminating PD from MSA-P and PSP: AUC = 0.99; Se = 90%; and Sp = 100% FA of SN, RD of SCP, and MCP in discriminating PD from MSA-P: AUC = 0.99; Se = 94%; and Sp = 100% FA of SN, AD of putamen in discriminating PD from PSP: AUC = 0.96; Se = 87%; and Sp = 100% FA of caudate, RD of MCP in discriminating MSA-P from PSP: AUC = 0.97; Se = 90%; and Sp = 100%	

Baudrexel et al., 2014 [83]	3 T	DTI/volumetry/FDG-PET	PD = 13; PSP = 8; MSA-P = 11; HC = 6 DD (y, M ± SD): PD = 6.4 ± 6.0; PSP = 2.6 ± 1.6; MSA-P = 3.6 ± 2.2	↑ MD in posterior putamen in MSA-P versus PD, PSP, and HC ↑ MD in anterior putamen in MSA-P versus PD ↓ putaminal volume in MSA-P versus PD, PSP, and HC	AUC/Se/Sp of posterior putamen MD in discriminating MSA-P from PD, PSP, and NC: 89%/72.7%/100% AUC/Se/Sp of putaminal volume in discriminating MSA-P from PD, PSP, and NC: 84%/54.5%/100%

Hess et al., 2014 [84]	3 T	DTI + volumetric	PD = 9; PSP = 5; CBS = 6; HC = 12 DD (y, M ± SD): PD = 8.6 ± 4.2; PSP = 3.8 ± 1.4; CBS = 5.5 ± 1.4	↓ thalamic volume in PSP and CBS versus PD and HC ↑ ADC in thalamus in PSP versus PD and HC	Not reported

Nilsson et al., 2007 [85]	3 T	Deterministic tractography	PD = 2; MSA = 4; PSP = 3; HC = 2 DD (y, range): PD = 2–10; MSA = 2–6; PSP = 2–6	↓ FA and ↑ ADC in MCP in MSA versus PSP, PD, and HC ↓ FA and ↑ ADC in SCP in PSP versus MSA, PD, and HC	Not reported

Surova et al., 2013 [86]	3 T	Deterministic tractography	PD = 10; MSA-P = 12; PSP = 16; HC = 16 DD [y, M (range)]: PD = 4.5 (2.0–7.5); MSA-P = 3.0 (2.2–5.0); PSP = 3.5 (2.2–4.0)	↑ MD, RD, and ↓ FA in CC in PSP versus PD and HC ↓ apparent area coefficient in CG and ↑ MD in CST in PSP versus PD ↑ RD in CST in MSA-P versus PD	AUC/Se/Sp of CG apparent area coefficient in differentiating PSP from PD: 0.88/87%/80% AUC/Se/Sp of CST MD in differentiating PSP from PD: 0.85/94%/80% AUC/Se/Sp of CC MD in differentiating PSP from PD: 0.85/81%/80%

Rosskopf et al., 2014 [87]	3 T	DTI/probabilistic tractography	PD = 15; PSP = 15 (7 PSP-RS; 8 PSP-P); HC = 18 DD [y, M (range)]: PD = 4 (1–7); PSP = 3 (2–5)	↓ FA in areas I, II, and III of CC in PSP versus PD and HC	Not reported

Surova et al., 2015 [88]	3 T	DTI/probabilistic tractography//morphometric and volumetric measurement	PSP = 27; MSA-P = 11; PD = 10; HC = 21 DD [y, median (IR)]: PSP = 3 (2–4); MSA-P = 3 (3–5); PD = 4 (2–7)	↑ MD in thalamus, ventral anterior, and ventral posterior thalamic nuclei and midbrain in PSP versus MSA-P, PD, and HC ↑ MD in pons and putamen in MSA-P versus PD and HC ↑ MD and ↓ FA of bilateral DRTT in PSP versus MSA-P, PD, and HC ↓ thalamus, putamen, and pallidus volumes and midbrain area in PSP versus MSA-P, PD, and HC in both cohorts ↓ putamen and pallidus volumes in MSA-P versus PD and HC	Se/Sp of MD of the thalamus, right DRTT, and midbrain in discriminating PSP from PD and MSA-P: 81–77%, 92–81%, and 81-81%, respectively

Meijer et al., 2015 [89]	3 T	DTI	PD = 30; aPS = 19 (12 MSA-P; 3 PSP; 3 DLB) DD [m, M ± SD]: PD = 21.6 ± 11.9; aPS = 28.4 ± 11.1	↑ MD in putamen, left SCP in MSA-P versus PD ↑ MD in midbrain and right SCP in PSP versus MSA-P, DLB, and PD	Diagnostic accuracy in discriminating aPS from PD = 75%

Eckert et al., 2004 [90]	1.5 T	MTI	PD = 15; PSP = 10; MSA = 12; HC = 20 DD (y, M ± SD): PD = 5.4 ± 4.0; PSP = 4.2 ± 2.7; MSA = 3.6 ± 1.6;	↓ MTR of globus pallidus in PSP versus PD, MSA and HC ↓ MTR of putamen in MSA versus PD and HC ↓ MTR in substantia nigra in PD, PSP, and MSA versus HC ↓ MTR in caudate and prefrontal WM in PSP versus HC	

von Lewinski et al., 2007 [91]	1 T	T2^*∗*^	PD = 88; MSA = 52 (47 MSA-P; 5 MSA-C); HC = 29 DD (y, M ± SD): MSA = 4.1 ± 2.0;	↓ T2^*∗*^ signal intensity putamen/caudate and putamen/thalamus in MSA versus PD and HC	Se/Sp signal intensity putamen/caudate in discriminating MSA from PD: 65%/95%

Gupta et al., 2010 [92]	1.5 T	SWI (semiquantitative hypointensity score)	PD = 11; PSP = 12; MSA-P = 12; HC = 11 DD (y, M ± SD): PD = 8.1 ± 3.9; PSP = 3.5 ± 3.9; MSA-P = 5.4 ± 3.3	↓ signal intensity of red nucleus, substantia nigra in PSP versus MSA-P PD and HC ↓ signal intensity in posterolateral putamen in PSP versus PD	Se/Sp of red nucleus hypointensity score in differentiating PSP from PD and from MSA-P: 66.7%/81.8%–66.7%/83.3% Se/Sp of putamen hypointensity score in differentiating PSP from PD: 50.0%/90.9%

Boelmans et al., 2012 [93]	1.5 T	Quantitative T2, T2^*∗*^, and T2′	PD = 30; PSP = 12; HC = 24 DD [y, M ± SD (range)]: PD = 9.7 ± 5.2 (0.6–22.7); PSP = 4.5 ± 3.7 (0.7–10.3)	↓ T2′ time in caudate, globus pallidus, and putamen in PSP versus PD and HC	Classification of linear discriminant analysis including basal ganglia and thalamus: 74.2%

Wang et al., 2012 [94]	1.5 T	SWI	PD = 16; MSA-P = 8; HC = 44 DD (y, M ± SD): PD = 2.5 ± 1.7; MSA-P = 2.3 ± 1.1	↑ (phase shift values) iron content in putamen and thalamus in MSA-P versus PD	Acc of high iron percentage in putamen in discriminating MSA-P from PD: 0.88

Han et al., 2013 [95]	3 T	SWI	PD = 15; PSP = 11; MSA-P = 12; HC = 20 DD (m, M ± SD): PD = 30.3 ± 19.10; PSP = 25.6 ± 11.57; MSA-P = 26.4 ± 6.24	↑ (phase shift values) iron content in the red nucleus, putamen, globus pallidus, and thalamus in PSP versus PD ↑ (phase shift values) iron content in the red nucleus and putamen in MSA-P versus PD ↑ (phase shift values) iron content in substantia nigra in PD versus MSA-P and HC	AUC of putamen in discriminating MSA-P from PSP and PD: 0.836 AUC of globus pallidus and thalamus in discriminating PSP from MSA-P and PD: 0.869 and 0.884, respectively

Lee et al., 2013 [96]	3 T	R2^*∗*^/volumetry	PD = 29; PSP = 13; MSA-P = 15 DD (m, M ± SD): PD = 29.41 ± 22.2; PSP = 27.62 ± 15.5; MSA-P = 22.73 ± 8.7;	↑ R2^*∗*^ in the putamen in MSA-P versus PD and HC ↑ R2^*∗*^ in globus pallidus and caudate in PSP versus PD and HC ↑ R2^*∗*^ in caudate nucleus in PSP versus MSA-P ↓ volume of caudate, putamen, globus pallidus, and thalamus in PSP and MSA-P versus PD and HC ↓ volume of globus pallidus in PSP versus MSA-P ↓ volume of putamen in MSA-P versus PSP	AUC of putaminal volume in discriminating MSA-P from PSP and PD: 0.832 AUC of globus pallidus volume in discriminating PSP from MSA-P and PD: 0.856

Yoon et al., 2015 [97]	3 T	SWI	PD = 30; MSA-P = 17 DD [y, M ± SD (range)]: PD = 6.07 ± 4.93 (1–21); MSA-P = 2.18 ± 1.19 (1–4.5)	↓ signal intensity of bilateral posterior halves, mean values of the anterior and posterior halves, and the dominant-side posterior half of the putamen in MSA-P versus PD	AUC of signal intensity of the dominant-side posterior half of the putamen in discriminating MSA-P from PD: 0.947

Davie et al., 1995 [98]	1.5 T	^1^H-MRS Single-voxel	PD = 9; MSA-P = 7; MSA-C = 5; HC = 9	↓ NAA/Cr and ↓ Cho/Cr in lenticular nucleus in MSA-P versus HC ↓ NAA/Cr in lenticular nucleus in MSA-C versus HC	

Federico et al., 1997 [99]	1.5 T	^1^H-MRS Single-voxel	PD = 8; PSP = 5; HC = 9 DD [y, M (range)]: PD = 7 (4–12); PSP = 5 (3–8)	↓ NAA/Cr and ↓ NAA/Cho in lenticular nucleus in PSP versus HC	Not reported

Federico et al., 1997 [100]	1.5 T	^1^H-MRS Single-voxel	PD = 12; PSP = 7; MSA = 7; HC = 10 DD [y, M ± SD (range)]: PD = 5.6 ± 2.6 (3–12); MSA = 3.7 ± 1.6 (3–6)	↓ NAA/Cho and ↓ NAA/Cr in lenticular nucleus in PSP and MSA versus HC	Not reported

Tedeschi et al., 1997 [101]	1.5 T	^1^H-MRS Single-voxel	PD = 10; PSP = 12; CBS = 9; HC = 11 DD (m, M ± SD): PD = 103 ± 9; PSP = 48 ± 9; CBS = 40 ± 5	↓ NAA/Cr in brainstem, centrum semiovale, and precentral cortices and ↓ NAA/Cho in lenticular nucleus in PSP versus HC ↓ NAA/Cr in centrum semiovale and ↓ NAA/Cho in lenticular nucleus and parietal cortex in CBS versus HC	Not reported

Federico et al., 1999 [102]	1.5 T	^1^H-MRS Single-voxel	PD = 19; PSP = 11; MSA = 14; HC = 12	↓ NAA/Cho in lenticular nucleus in PSP and MSA versus PD and HC ↓ NAA/Cr in lenticular nucleus in PD, PSP, and MSA versus HC and in MSA versus PD	Not reported

Abe et al., 2000 [103]	1.5 T	^1^H-MRS Single-voxel	PD = 23; PSP = 12; MSA = 18; CBS = 19; VP = 10; HC = 20 DD (y, M ± SD): PD = 3.6 ± 1.5; PSP = 3.9 ± 1.6; MSA = 4.0 ± 1.5; CBS = 3.6 ± 1.7; VP = 3.4 ± 1.7	↓ NAA/Cr of frontal cortex in PSP, MSA, CBD, and VP versus HC ↓ NAA/Cr of putamen in PSP, MSA, CBD, and PD versus HC ↓ NAA/Cr of frontal cortex and putamen in CBD versus PD, MSA, and VP ↓ NAA/Cr of putamen in PSP versus VP and MSA	Not reported

Clarke and Lowry, 2000 [104]	1.5 T	^1^H-MRS Single-voxel	PD = 6; MSA = 6; HC = 6	↓ NAA/Cho and ↑ Cho/Cr in lenticular nucleus in PD versus HC	Not reported

Watanabe et al., 2004 [105]	3 T	^1^H-MRS Single-voxel	24 MSA = 24 (13 MSA-C and 11 MSA-P); PD = 11; HC = 18 DD (y, M ± SD): MSA = 3.7 ± 2.4; PD = 4.4 ± 2.2	↓ NAA/Cr of pontine base in all MSA types and of putamen in MSA-P versus HC ↓ NAA/Cr of pontine base and putamen in MSA-P versus PD	Not reported

Vasconcellos et al., 2009 [106]	1.5 T	^1^H-MRS (Single-voxel)	PD = 12; PSP = 11; MSA-P = 7; HC = 10 DD (y, M ± SD): PD = 8.5 ± 3.5; PSP = 6.6 ± 3.1; MSA-P = 8.0 ± 2.0	↓ NAA/Cr of lenticular nucleus in PSP versus PD and HC ↓ NAA/Cr of the hippocampus in PSP versus HC ↓ NAA/Cho of the midbrain in PSP versus MSA-P and HC	Not reported

Guevara et al., 2010 [107]	1.5 T	^1^H-MRS (Multi- and single-voxel)	PD = 11; PSP = 13; MSA-P = 11; MSA-C = 6; HC = 18 DD [y, M ± SD (range)]: PD = 6.9 ± 2.1 (3.9–10.5); PSP = 5.1 ± 2.1 (2.3–10.5); MSA-P = 4.7 ± 2.6 (2.0–9.6); MSA-C = 6.1 ± 2.1 (4.0–9.0)	↓ NAA of putamen and pallidum in MSA-P and PSP versus PD and HC ↓ NAA of pallidum in PSP versus MSA-P	Not reported

Zanigni et al., 2015 [108]	1.5 T	^1^H-MRS (Single-voxel)	PSP-RS = 21; MSA-P = 7; MSA-C = 8; PD = 21; HC = 14 DD [y, median (range)]: PSP-RS = 4 (1–11); MSA-P = 3(0.2–7); MSA-C = 6 (3–13); PD = 3 (1–15)	↓ cerebellar NAA/Cr and NAA/mI ratios in aPS versus PD and HC and in MSA-C versus PSP-RS, MSA-P, and PD (*p* < 0.01) ↓ cerebellar NAA/Cr ratio in PSP-RS versus PD and NC (*p* < 0.05) and ↓ cerebellar NAA/mI in PSP-RS versus NC (*p* < 0.01)	Se/Sp of cerebellar NAA/Cr ratio value in discriminating PD from aPS: 100% and 64%.

Acc/Se/Sp: accuracy/sensitivity/specificity; T: Tesla; PSP: Progressive Supranuclear Palsy; PD: Parkinson's disease; HC: healthy controls; DD: disease duration; y: years; M: mean; SD: standard deviation; MSA: Multiple System Atrophy; MSA-P: parkinsonian variant of MSA; MCP: middle cerebellar peduncle; SCP: superior cerebellar peduncle; MRPI: MR parkinsonism index; MSA-C: cerebellar variant of MSA; PSP-RS: PSP-Richardson's syndrome; PSP-P: PSP-parkinsonism; m: months; FA: fractional anisotropy; MD: mean diffusivity; TIV: total intracranial volume; SEM: standard error mean; CBS: corticobasal syndrome; CC: corpus callosum; WM: white matter; DWI: diffusion-weighted imaging; rADC: regional ADC; rTrace(D): trace of diffusion tensor; ADC_ave_: ADC average; HSR: hemispheric symmetry ratio; ADC: apparent diffusion coefficient; GRE: gradient echo; DTI: diffusion tensor imaging; IR: interquartile range; aPS: atypical parkinsonian syndromes; DLB: dementia with Lewy bodies; MTRI: magnetization transfer imaging; MTR: magnetization transfer; SWI: susceptibility-weighted imaging; AUC: area under the curve; ^1^H-MRS: proton magnetic resonance spectroscopy; NAA: N-acetyl-aspartate; Cr: creatine; Cho: choline; VP: vascular parkinsonism.

**Table 3:** Anatomical distribution of brain alterations in multiple system atrophy (MSA), in the cerebellar (-C) and parkinsonian (-P) MSA variants, in progressive supranuclear palsy (PSP), and in corticobasal syndrome (CBS) (results of those studies focused on the differential diagnosis between Parkinson's disease and atypical parkinsonian syndromes).

	MSA	MSA-C	MSA-P	PSP	CBS
*Basal ganglia*					

*Putamen*	↓ volume^*∗*^ [53] ↑ ADC^∧^ [73] ↓ MTR^*∗*†^ [90] ↓ T2^*∗*^ signal intensity^*∗*†^ [91] ↓ NAA/Cr^†^ [103]		↓ volume^*∗*∧†^ [55, 60, 83, 86, 96] ↑ ADC^*∗*°§†^ [61, 62, 64, 66, 73, 75, 77] ↑ rTrace(D)^*∗*§†^ [63, 65, 68, 71] ↓ FA^*∗*^ [75] ↑ MD^*∗*∧†^ [83, 86, 89] ↑ phase shift values^*∗*^ [94, 95] ↑ R2^*∗*^ signal intensity^*∗*†^ [96] ↓ SWI signal intensity^*∗*^ [97] ↓ NAA/Cr^*∗*†^ [105] ↓ NAA^*∗*†^ [107]	↓ volume^*∗*°†^ [54, 55, 60, 86, 96] ↑ ADC^*∗*^ [62, 66, 70, 72] ↓ T2′ time^*∗*†^ [93] ↑ MD^*∗*^ [54] ↓ SWI signal intensity^*∗*^ [92] ↑ phase shift values^*∗*^ [95] ↓ NAA/Cr^#†^ [103]↓ NAA^*∗*†^ [107]	↑ ADC^*∗*^ [70] ↓ NAA/Cr^*∗*#†^ [103]

*Globus pallidus*			↓ volume^*∗*†^ [60, 86, 96] ↑ rTrace(D)^*∗*^ [63] ↑ ADC^*∗*§^ [66, 73] ↓ NAA^*∗*†^ [107]	↓ volume^*∗*°†^ [54, 60, 86, 96] ↑ ADC^*∗*#†^ [62, 66, 73] ↑ MD^*∗*^ [54, 79] ↓ MTR^*∗*#†^ [90] ↓ T2′ time^*∗*†^ [93] ↑ phase shift values^*∗*^ [95] ↑ R2^*∗*^ signal intensity^*∗*†^ [96] ↓ NAA^*∗*°†^ [107]	

*Lenticular nucleus*	↓ NAA/Cr^*∗*†^ [100, 102] ↓ NAA/Cho^*∗*†^ [100, 102]	↓ NAA/Cr^†^ [98]	↓ NAA/Cr^†^ [98] ↓ Cho/Cr^†^ [98]	↓ NAA/Cr^*∗*†^ [99–102, 109] ↓ NAA/Cho^*∗*†^ [99, 100, 102]	↓ NAA/Cho^†^ [101]

*Caudate*			↓ volume^*∗*†^ [55, 96] ↑ ADC^*∗*§^ [62, 66, 73] ↑ rTrace(D)^*∗*^ [63]	↓ volume^*∗*†^ [55, 96] ↑ ADC^*∗*#†^ [62, 66, 73] ↓ MTR^†^ [90] ↓ T2′ time^*∗*†^ [93] ↑ R2^*∗*^ signal intensity^*∗*°†^ [96]	

*Nucleus accumbens*				↓ volume^*∗*^ [54]	

*Thalamus*			↑ ADC^*∗*^ [66] ↑ phase shift values^*∗*^ [94] ↓ volume^*∗*^ [96]	↓ volume^*∗*°†^ [54, 60, 84, 86, 96] ↑ ADC^*∗λ*†^ [66, 78] ↑ MD^*∗*°†^ [54, 86] ↑ phase shift values^*∗*^ [95]	↓ volume^*∗*†^ [84]

*Supratentorial compartment*					

Whole brain				↓ volume^*∗*†^ [56] ↑ MD^*∗*^ [54] ↓ FA^*∗*^ [54]	↑ ADC^*∗*∧^ [70] ↓ HSR^*∗*∧^ [70]

*Frontal lobe*	↓ NAA/Cr^†^ [103]			↓ volume^*∗*†^ [56, 59] ↑ MD^*∗*^ [54] ↓ FA^*∗*^ [54] ↓ MTR^†^ [90] ↓ precentral cortices NAA/Cr^†^ [101] ↓ NAA/Cr^†^ [103]	↑ ADC precentral gyrus^∧^ [78] ↓ NAA/Cr^*∗*#†^ [103]

*Parietal lobe*				↑ MD^*∗*^ [54] ↓ FA^*∗*^ [54]	↓ volume^∧†^ [57] ↑ ADC postcentral gyrus^∧^ [78] ↓ NAA/Cho^†^ [101]

*Occipital lobe*					↓ volume^∧†^ [57]

*Corpus Callosum*				↑ ADC in CC1^*∗*†^ and CC2^*∗*^ [76] ↓ FA in CC1^†^ and CC2^*∗*†^ [76] ↑ MD^*∗*†^ [86] ↑ RD^*∗*†^ [86] ↓ FA^*∗*†^ [86, 87]	↓ area^∧†^ [57] ↑ MD^*∗*†^ [80] ↓ FA^*∗*†^ [80]

*Cingulate gyrus *				↑ ADC^*λ*^ anterior CG [78] ↓ apparent area coefficient^*∗*^ [86]	

*Hippocampus *			↓ volume^*∗*†^ [60]	↓ volume^*∗*†^ [60] ↓ NAA/Cr^†^ [107]	

*Centrum semiovale*				↓ NAA/Cr^†^ [101]	

*Lateral ventricles*				↑ volume^*∗*†^ [54, 56, 60]	

*3rd ventricle*				↑ volume^*∗*†^ [59, 60]	

*4th ventricle*			↑ volume^*∗*†^ [60]	↑ volume^*∗*†^ [60]	

*White matter tracts*					

*CST*			↑ RD [86]	↑ MD^*∗*^ [86]	

*DRTT*				↑ MD^*∗*°†^ [88] ↓ FA^*∗*°†^ [88]	

*Infratentorial compartment*					

*Whole *		↓ volume^†^ [54] ↑ MD^*∗*∧†^ [81]	↓ volume^†^ [54] ↑ MD^*∗*∧†^ [81]	↓ volume^*∗λ*†^ [55, 57, 60] ↑ MCP/SCP^*∗*°^ [37, 54] ↓ M/P^*∗*°^ [47, 50, 52] ↑ P/M^*∗*°^ [37, 54] ↑ MRPI^*∗*°^ [37, 50, 52, 54] ↑ MD^*∗*†^ [54, 81] ↓ FA^*∗*^ [54]	

*Midbrain*			↓ area^*∗*§^ [49] ↓ volume^†^ [59]	↓ area^*∗*°§†^ [37, 46, 47, 49, 52, 54, 88] ↓ volume^*∗*°†^ [59] ↑ ADC^*∗*#†^ [73] ↑ MD^*∗*°†^ [88, 89] ↓ FA^*∗*^ [54] ↓ NAA/Cho^°†^ [106]	

*Substantia Nigra*	↓ FA^*∗*^ [53] ↓ MTR^†^ [90]			↑ MD^*∗*°^ [79] ↓ MTR^†^ [90] ↓ SWI signal intensity^*∗*°†^ [92]	

*Red nucleus *			↑ phase shift values^*∗*^ [95]	↑ phase shift values^*∗*^ [95] ↓ SWI signal intensity^*∗*°†^ [92]	

*Pons*	↓ anteroposterior diameter^*∗*^ [53] ↓ volume^*∗*^ [53] ↑ ADC^*∗*∧†^ [73] ↓ NAA/Cr^†^ [105]	↓ area^*∗*∧^ [49] ↑ MD^*∗*∧^ [74]	↓ area^*∗*∧^ [37, 47, 49] ↓ volume^*∗*†^ [59] ↑ ADC^*∗*∧^ [55, 59, 75] ↓ FA^*∗*^ [75] ↑ MD^*∗*†^ [88] ↓ NAA/Cr^*∗*^ [105]	↓ area^*∗*^ [46, 49, 52, 54]	

*Brainstem*		↓ volume^†^ [55] ↑ MD^*∗*∧†^ [81]	↓ volume^*∗*†^ [55, 60] ↑ MD^*∗*∧†^ [81]	↑ MD^*∗*†^ [54, 55, 81] ↓ FA^*∗*^ [54] ↓ volume^*∗*^ [54] ↓ NAA/Cr^†^ [101]	

*SCP*		↓ diameter^*∗*^ [49]	↑ MD^*∗*^ [89]	↓ width^*∗*°^ [37] ↓ diameter^*∗*°§^ [49, 52, 54] ↓ volume^*∗*#†^ [59] ↑ ADC^*∗*°*λ*†^ [69, 70, 73, 78] ↑ MD^*∗*#°^ [54, 74, 89] ↓ FA^*∗*^ [54, 74]	

*MCP*	↓ mean width^*∗*^ [53] ↑ MD^*∗*∧^ [53, 74] ↓ FA^*∗*∧†^ [53, 74, 85] ↑ ADC^*∗*∧†^ [73, 85]	↓ diameter^*∗*∧^ [49] ↑ trace(D)^°†^ [71]	↓ width^*∗*°^ [37, 48] ↓ diameter^*∗*^ [49] ↑ ADC^*∗*∧†^ [48, 67, 77]	↓ diameter^*∗*^ [49, 52, 54] ↓ FA^*∗*#†^ [85] ↑ ADC^*∗*#†^ [85]	

*Cerebellum *	↓ volume^*∗*^ [53] ↑ ADC^*∗*∧†^ [73] ↓ FA^*∗*^ [53]	↓ volume^†^ [54] ↑ trace(D)^°†^ [71]	↓ volume^*∗*†^ [54, 59, 60] ↑ ADC^*∗*^ [75] ↓ FA^*∗*^ [75]	↓ volume (cortex)^*∗*†^ [60]	

*Cerebellar vermis*		↑ MD^*∗*°†^ [81]	↑ MD^*∗*†^ [81]	↑ MD^*∗*†^ [81]	

*Cerebellar hemispheres*		↑ MD^*∗*∧†^ [81] ↓ NAA/Cr^*∗*∧°^ [108]	↑ MD^*∗*∧†^ [81]	↑ MD^*∗*^ [54] ↓ NAA/Cr^*∗*†^ [108] ↓ NAA/mI^†^ [108]	

ADC: apparent diffusion coefficient; MTR: magnetization transfer; rTrace(D): trace of diffusion tensor; NAA: N-acetyl-aspartate; CR: creatine; FA: fractional anisotropy; MD: mean diffusivity; SWI: susceptibility weighted imaging; HSR: hemispheric symmetry ratio; Cho: choline; CC: corpus callosum; RD: radial diffusivity; CST: corticospinal tract; DRTT: dentatorubrothalamic-tract; MCP: middle cerebellar peduncle; SCP: superior cerebellar peduncle; M: midbrain; P: pons; MRPI: MR parkinsonism index.

^*∗*^versus PD; ^∧^versus PSP; °versus MSA-P; ^§^versus MSA-C; ^#^versus MSA; ^*λ*^versus CBS; ^†^versus HC.
